# Cytoresistance after acute kidney injury is limited to the recovery period of proximal tubule integrity and possibly involves Hippo‐YAP signaling

**DOI:** 10.14814/phy2.13310

**Published:** 2017-06-14

**Authors:** Takamasa Iwakura, Yoshihide Fujigaki, Tomoyuki Fujikura, Takayuki Tsuji, Naro Ohashi, Akihiko Kato, Hideo Yasuda

**Affiliations:** ^1^ Internal Medicine IDivision of NephrologyHamamatsu University School of MedicineHamamatsuJapan; ^2^ Department of MedicineTeikyo University School of MedicineTokyoJapan; ^3^ Blood Purification UnitHamamatsu University School of MedicineHamamatsuJapan

**Keywords:** Cytoresistance, G1 arrest, proximal tubule, Survivin, YAP

## Abstract

Rat proximal tubule (PT) cells that have recovered from severe acute kidney injury induced by uranyl acetate (UA) develop cytoresistance to subsequent UA treatments. We reported that enhanced G1 arrest might contribute to cytoresistance. Herein, we examined these mechanisms by investigating Yes‐associated protein (YAP), a regulator of cell number, and survivin, a downstream mediator of YAP that inhibits apoptosis. Rats pretreated with saline (vehicle group) or UA (AKI group) were injected with UA 2 weeks, 2 months, or 6 months after treatment. Cytoresistance, evaluated by serum creatinine, was observed at 2 weeks, was attenuated at 2 months, and was lost at 6 months in the AKI group. Based on immunohistochemistry, overexpressed YAP/survivin in PT cells and an increased number of PT cells was found before the second insult at 2 weeks, regressed gradually, and returned to a normal value by 6 months in the AKI group. Cell cycle status, assessed by flow cytometry, was equivalent in all groups before the second insult. However, early G1 phase (cyclin D1−) and p27+ PT cells increased in the AKI group compared to those in the vehicle group until 2 months, but were comparable to those in the vehicle group at 6 months. p21+ PT cells increased at 2 weeks, but normalized by 2 months. Thus, PT cells that have recovered from AKI transiently overexpress YAP/survivin, probably inhibiting apoptosis and resulting in acquired cytoresistance. This effect occurs until PT remodeling is complete, subceullular PT integrity is restored, and cell numbers are normalized.

## Introduction

Animals that have recovered from acute kidney injury (AKI) can develop cytoresistance to subsequent nephrotoxin insults, and this phenomenon is termed acquired resistance to rechallenge injury (Honda et al. 1987). This phenomenon of resistance to injury after prior exposure and subsequent recovery has been shown by many investigators in diverse forms of injury, including ischemia/reperfusion (Nowak et al. 2004; Kapitsinou and Haase 2015), oxidants (Nowak et al. 2012), and alkylating agents (Vaidya et al. 2003; Korrapati et al. 2005, 2006). We have examined the potential mechanisms of acquired resistance in cisplatin‐ and uranyl acetate (UA)‐induced AKI models and identified a variety of associated factors (Furuya et al. 1997; Mizuno et al. 1997; Sano et al. 2000; Miyaji et al. 2001; Sun et al. 2010, 2011; Fujikura et al. 2013; Iwakura et al. 2016). Recently, we demonstrated that acquired resistance in UA‐induced AKI in rats is associated with enhanced G1 arrest in proximal tubule (PT) cells, which facilitates DNA repair and evasion of apoptosis (Iwakura et al. 2016), and is associated with the modulation of cell cycle‐mediated factors such as p21 and p27.

It is known that this acquired resistance to rechallenge injury can last for limited periods, for example, for 6 months in a gentamicin‐induced AKI model (Elliott et al. 1982). In rats with UA‐induced AKI, we found that renal function, evaluated by serum creatinine (SCr), returned to baseline levels and the dedifferentiated phenotype in the PT also returned to a differentiated phenotype (i.e., re‐expression of megalin and loss of dedifferentiation markers such as vimentin and paired box gene‐2 [Pax‐2]) by day 14 after insult, when cytoresistance to rechallenge injury was observed. However, PT hyperplasia and the overexpression of p21 and p27 in PT cells were sustained at that time. Subsequently, it was assumed that these changes gradually normalize and that PT cells can regain their subcellular integrity.

Accordingly, we hypothesized that cytoresistance in PT cells would be lost with the normalization of tubule integrity after injury, and that this might be associated with Yes‐associated protein (YAP), an effector protein of the Hippo signaling pathway, and a regulator of organ cell numbers (Yu and Guan 2013). In the present study, we examined the relationship between PT cell cytoresistance and the expression of YAP and survivin, a downstream mediator of YAP and a member of the inhibitor of apoptosis family (Altieri 2010), in association with changes in PT integrity over time after injury.

## Materials and Methods

### Rats

Male Sprague Dawley rats weighing 180–250 g (SLC Co., Shizuoka, Japan) were provided standard rat chow and drinking water ad libitum. The experimental protocol was approved by the Ethics Review Committee for Animal Experimentation of Hamamatsu University School of Medicine.

### Reagents

Uranyl acetate (UA) dihydrate (purity >98.0%) was purchased from Fluka (Buchs, Switzerland). Collagenase type II was from Worthington Biochemical Corp. (Lakewood, NJ). Percoll was purchased from GE Healthcare UK, Ltd. (Little Chalfont, Buckinghamshire, UK). Trypan blue solution, propidium iodide, Hoechst 33342, and pyronin Y were purchased from Sigma‐Aldrich Co. (St. Louis, MO). Hank's balanced salt solution (HBSS) was from Invitrogen (Carlsbad, CA). Can Get Signal^®^ solution B was from Toyobo Life Science Department (Osaka, Japan). Citrate buffer solution was from Mitsubishi Chemical Medience (Tokyo, Japan). Histofine Antigen Retrieval Solution pH9^®^ and Histofine MAX PO kit were from Nichirei Bioscience (Tokyo, Japan). ApopTag^®^ Plus In Situ Apoptosis Detection Kit was from Chemicon‐Millipore (Temecula, CA). Picrosirius red stain kit was from Polysciences, Inc. (Warrington, PA). The antibodies listed in Table 1 were used as primary antibodies. Alexa Fluor^®^ 633‐conjugated donkey anti‐goat IgG (Invitrogen), Alexa Fluor^®^ 546‐conjugated goat anti‐rabbit IgG (Invitrogen), Alexa Fluor 488‐conjugated donkey anti‐mouse IgG (Invitrogen), and Histofine Simple Stain Max PO (Nichirei Bioscience) were used as secondary antibodies.

**Table 1:** Primary antibodies, their dilutions and suppliers

Antibody	Dilution	Suppliers
Immunocytochemistry		
Rabbit polyclonal anti‐Cdt1 (H‐300)	1:100	Santa Cruz Biotechnology
Goat polyclonal anti‐megalin (P‐20)	1:200	Santa Cruz Biotechnology
Immunohistochemistry		
Rabbit polyclonal anti‐YAP (#4192)	1:200	Cell signaling
Rabbit monoclonal anti‐survivin (#2808)	1:200	Cell signaling
Rabbit monoclonal anti‐Ki67 (SP6)	1:200	Thermo Fisher Scientific
Rabbit monoclonal anti‐cyclin D1 (SP4)	1:200	Thermo Fisher Scientific
Mouse monoclonal anti‐p21 (F‐5)	1:200	Santa Cruz Biotechnology
Rabbit polyclonal anti‐p27 (M‐197)	1:200	Santa Cruz Biotechnology
Goat polyclonal anti‐megalin (P‐20)	1:200	Santa Cruz Biotechnology
Mouse monoclonal anti‐vimentin (V9)	1:200	Sigma‐Aldrich Co
Rabbit polyclonal anti‐Pax2 (71‐6000)	1:100	Zymed
Mouse polyclonal anti‐Kim‐1 (AF1817)	1:300	R&D Systems
Rabbit monoclonal anti‐phospho‐histone H3 at serine 10 (D2C8)	1:400	Cell Signaling
Mouse monoclonal anti‐αSMA (M0851)	1:100	Dako

### Experimental protocol

As reported previously (Fujigaki et al. 2009; Sun et al. 2010), it was shown that in a UA‐induced AKI model, SCr increased and peaked at days 5–7, and returned to normal levels by day 14. Histologically, PT injury, with apoptotic and necrotic cells, peaked at day 5 and recovery occurred by day 14. One hundred and forty‐four rats were treated intravenously with saline (vehicle group) or 1 mg/kg of UA dissolved in saline (AKI group) as the first treatment. Subsequently, 2 weeks, 2 months, or 6 months after the first treatment, rats in the vehicle group (*N* = 36) or the AKI group (*N* = 36) were anesthetized via intraperitoneal injection with ketamine (75 mg/kg) and xylazine (10 mg/kg), and euthanized for the isolation of tubular cells and for histological examination and evaluation of renal function (*N* = 6 at each time point for each procedure). Two weeks, 2 months, or 6 months after the first treatment, rats in the vehicle group (*N* = 36) or the AKI group (*N* = 36) were injected with 1 mg/kg of UA intravenously as the second treatment. Animals were euthanized 5 and 14 days after the second insult for histological examination and evaluation of renal function (*N* = 6 at each time point). Blood samples were collected from the aorta at each time point and SCr concentration was measured by enzymatic assays (Falco SD, Kyoto, Japan).

### Histological examination

After a brief flush with phosphate‐buffered saline, both kidneys were dissected, bisected along their longitudinal axis, fixed with 4% paraformaldehyde, and embedded in paraffin. Sections of 3 μm thickness were examined. Immunohistochemical analysis was performed according to standard protocols described in detail previously (Iwakura et al. 2014). Sections were incubated with the primary antibodies listed in Table 1 and were reacted with Histofine Simple Stain MAX PO and visualized using a peroxidase–diaminobenzidine system.

Apoptosis was assessed by the terminal uridine nick‐end labeling (TUNEL) technique using the ApopTag^®^ Plus In Situ Apoptosis Detection Kit (Sun et al. 2010).

Tubulointerstitial fibrosis was evaluated by Masson's trichrome staining and picrosirius red using a picrosirius red stain kit that stains type I and III collagens.

Double immunofluorescent staining of phosphohistone H3 at serine 10 (PhH3s10) and alpha smooth muscle actin (αSMA) was performed. Sections fixed with 4% paraformaldehyde were incubated with both antibodies and subsequently with Alexa Fluor^®^ 546‐conjugated goat anti‐rabbit IgG and Alexa Fluor^®^ 488‐conjugated donkey anti‐mouse IgG.

### Isolation of PT and distal tubule (DT) cells

Renal tubular cells were isolated by collagenase (type II) perfusion and separated into PT and DT cell fractions by Percoll density gradient centrifugation as described by Lash et al. with slight modifications (Lash et al. 2001; Iwakura et al. 2014). Trypan blue exclusion was used to determine the percentage of viable cells present in the cell suspensions.

### Cell cycle analysis of isolated PT cells

As described previously (Iwakura et al. 2014), freshly isolated PT cells were permeabilized with Triton X‐100 and incubated with propidium iodide solution. After incubation, the DNA content was measured using an Epics XL flow cytometer (Beckman Coulter, Brea, CA). To separate cells in G1 phase from cells in G0 phase, freshly isolated PT cells, fixed with ice‐cold 70% ethanol, were incubated with Hoechst 33342/pyronin Y solution, and the DNA or RNA content was measured using a FACSAria^™^ cell sorter (Crissman et al. 1985) (BD Biosciences, San Jose, CA).

### Immunocytochemistry

To discriminate PT cells from DT cells, isolated PT and DT cells were fixed with 2% paraformaldehyde on a glass slide and incubated with goat anti‐megalin IgG (a PT brush border marker) with Can Get Signal^®^ solution B and then with Alexa Fluor^®^ 633‐conjugated donkey anti‐goat IgG. To discriminate G1 phase cells from G0 phase cells, isolated PT cells, permeabilized with 0.5% Triton X‐100, were incubated with rabbit anti‐Cdt1 IgG (G1 phase marker) and then with Alexa Fluor 546‐conjugated goat anti‐rabbit IgG. For nuclear staining, cells were incubated with 4′,6‐diamidino‐2‐phenylindole. The cells were then observed with a confocal fluorescence microscope (FV1000, Olympus, Tokyo, Japan).

### Morphometric analysis

Assessment of the purity of isolated PT cells, the percentage of Cdt1+ cells among the PT cells, and morphometric analysis of the renal sections were performed as described previously (Iwakura et al. 2014). Necrotic tubules were defined as described previously (Sun et al. 2010). The mean number of PT cells per tubule was calculated in 100 cross‐sections of the PT in the cortex and outer stripe of outer medulla (OSOM) at a magnification of 400× (Iwakura et al. 2014). The numbers of YAP+, survivin+, cyclin D1+, Ki67+, p21+, p27+, megalin+, vimentin+, Pax‐2+, kidney injury molecule‐1 (Kim‐1)+, PhH3s10+, and TUNEL+ cells in PTs and necrotic PTs were counted in 20 randomly selected fields of the cortex and OSOM at a magnification of 400× (Iwakura et al. 2014, 2016). The mean score in each rat represented the average number of PT cells and PTs per field. The degree of tubulointerstitial fibrosis, evaluated by Masson's trichrome staining, was assessed using a point‐counting method with 10 randomly selected fields of the cortex and OSOM at a magnification of 100×. The percentage of tubulointerstitial fibrosis, evaluated using the picrosirius red stain kit, was quantitatively assessed using a semiautomatic image analysis system with Image Pro Plus software (Media Cybernetics, GA) (Isobe et al. 2016). The mean score in each rat represented the average percent fibrotic area per field.

### Statistical analysis

All values were expressed as the mean ± SD. Differences between three or more groups were examined for statistical significance by using ANOVA, followed by Tukey's post hoc test. Differences between AKI group and vehicle group at the same time point were assessed using an unpaired *t*‐test (Prism 6; GraphPad Software, San Diego, CA). A *P* < 0.05 was accepted as statistically significant.

## Results

### Limited period of acquired resistance to rechallenge injury

**Figure 1:**
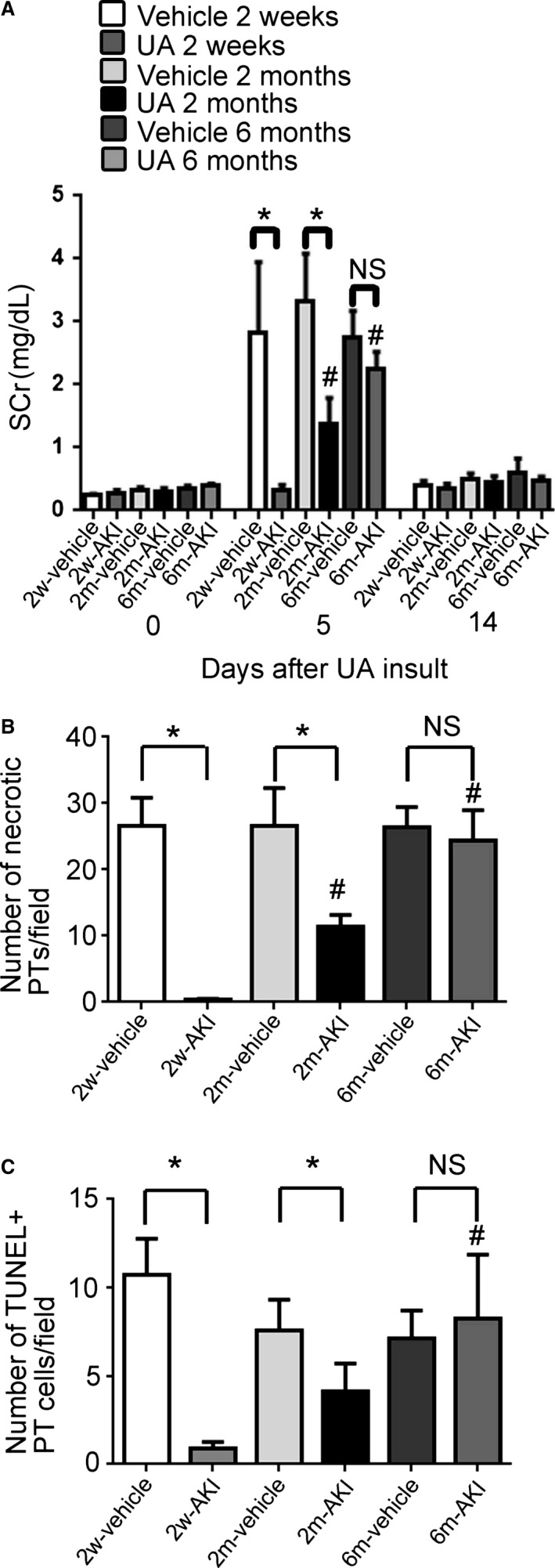
Changes in extent of acquired resistance after rechallenge injury. Serial changes in serum creatinine (SCr) levels (A). Morphometric analysis of the number of necrotic proximal tubules (PTs) (B) or TUNEL+ PT cells (C) 5 days after the second insult. Data represent the mean ± SD of six rats. *P < 0.05, #*P* < 0.05 versus rats 2 weeks after first treatment. NS, not significant; SCr, serum creatinine.

Immediately before the second insult (on day 0), SCr levels were equivalent in all groups (Fig. 1A). Following the second insult at any time point, SCr increased significantly at day 5 in the vehicle groups (Fig. 1A). However, following a second insult 2 weeks after the first treatment in the AKI group (2w‐AKI group), SCr did not increase. Following a second insult 2 months after the first treatment in the AKI group (2m‐AKI group), SCr increased significantly at day 5, but this value was lower than that in the vehicle group. Following a second insult 6 months after the first treatment in the AKI group (6m‐AKI group), SCr levels were comparable to those in the 6 m vehicle group. Fourteen days after the second insult, SCr returned to baseline levels (at day 0) in all groups (Fig. 1A).

Based on histological examinations, necrotic PTs were not found at day 0 for all groups; however, TUNEL+ PT cells were sparsely distributed (at day 0) in the 2w‐AKI group (data not shown). The number of necrotic PTs and TUNEL+ PT cells at day 5 after the second insult was significantly lower in the 2w‐ and 2m‐AKI groups than in the 2w‐ and 2m‐vehicle groups, respectively; however, after the second insult in the 6m‐AKI group, these values were comparable to those in the 6m‐vehicle group (Fig. 1B and C).

### Regression of cell number and YAP/survivin expression in PT cells after the induction of AKI

**Figure 2:**
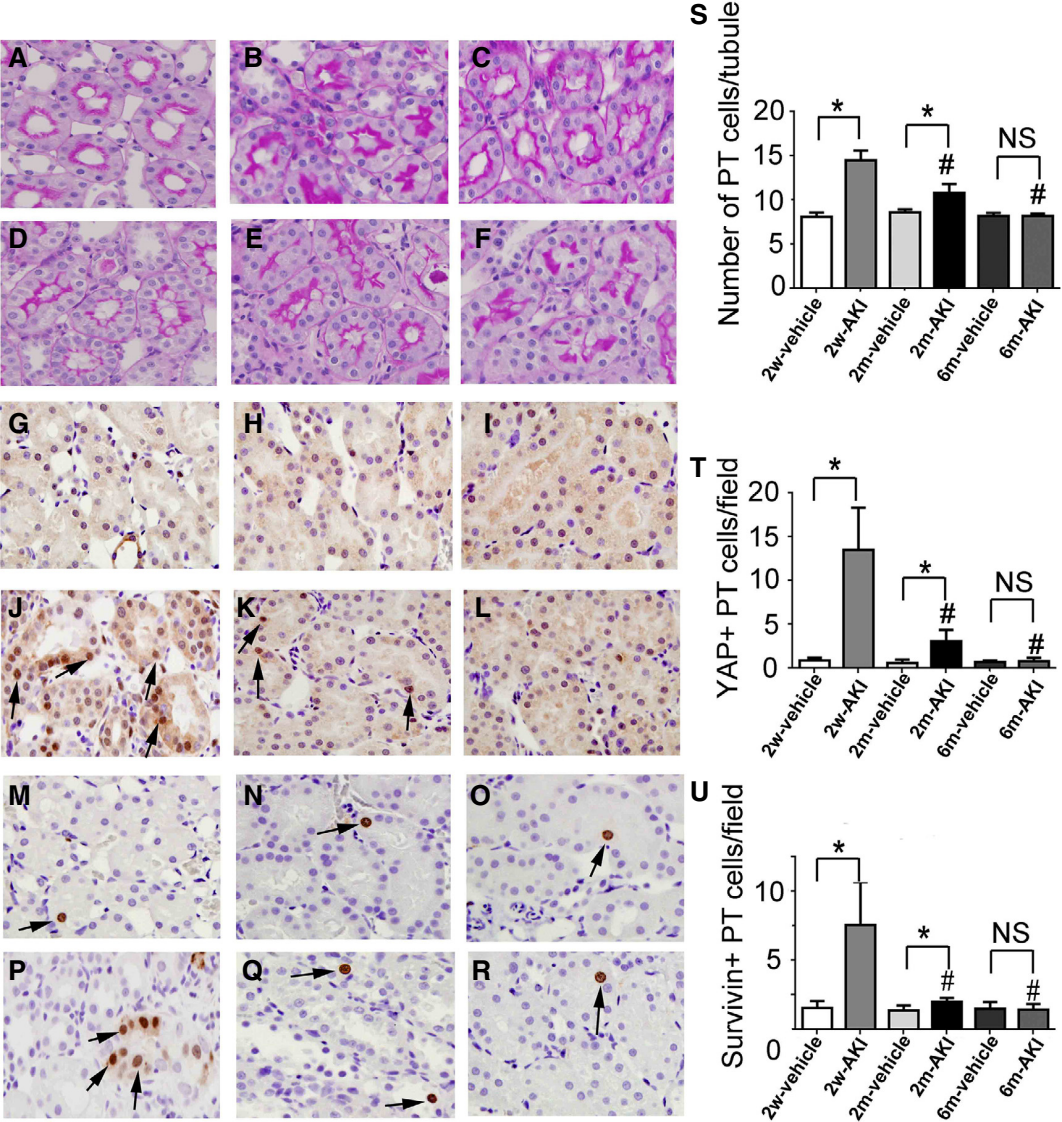
Changes in the expression of YAP/survivin in PT cells. Photomicrographs of periodic acid–Schiff‐stained renal sections from rats pretreated with vehicle (A–C) or with 1 mg/kg of uranyl acetate (UA) (D–F) for 2 weeks (A, D), 2 months (B, E), and 6 months (C, F) after treatment. Photomicrographs of YAP‐immunostained (arrows) renal sections from rats pretreated with vehicle (G–I) and with 1 mg/kg of UA (J–L) for 2 weeks (G, J), 2 months (H, K), and 6 months (I, L) after treatment. Photomicrographs of survivin‐immunostained (arrows) renal sections from rats pretreated with vehicle (M–O) and with 1 mg/kg of UA (P–R) for 2 weeks (M, P), 2 months (N, Q), and 6 months (O, R) after treatment. (S) Temporal changes in the number of cells per cross‐sectional PT. (T) Temporal changes in the number of YAP+ PT cells per field. (U) Temporal changes in the number of survivin+ PT cells per field. Original magnification, 400×. Data represent the mean ± SD of six rats. *P < 0.05, #*P* < 0.05 versus rats 2 weeks after first treatment. NS, not significant.

The number of cells per cross‐sectional PT at day 0 in the 2w‐ and 2m‐AKI groups was higher than that in the 2w‐ and 2m‐vehicle groups, respectively. The number of PT cells at day 0 in the AKI groups gradually decreased from 2 weeks to 6 months after treatment, and that in the 6m‐AKI group became comparable to that in the 6m‐vehicle group (Fig. 2A–F and S). This indicates that the hyperplastic state in the PT after recovery from the acute tubular injury returned to basal conditions by 6 months.

Next, the nuclear staining pattern of YAP and survivin was determined immunohistochemically in tubular cells (Fig. 2G–R). The number of YAP+ and survivin+ PT cells at day 0 in the 2w‐ and 2m‐AKI groups was higher than that in the 2w‐ and 2m‐vehicle groups, respectively. This number gradually decreased in the AKI group and became comparable to that in vehicle group at 6 months (Fig. 2G–L and T, Fig. 2M–R and U). These results could indicate that YAP/survivin overexpression in the PT is closely associated with the increased number of PT cells.

### Normalization of cell cycle status and CDKI expression after AKI

**Figure 3:**
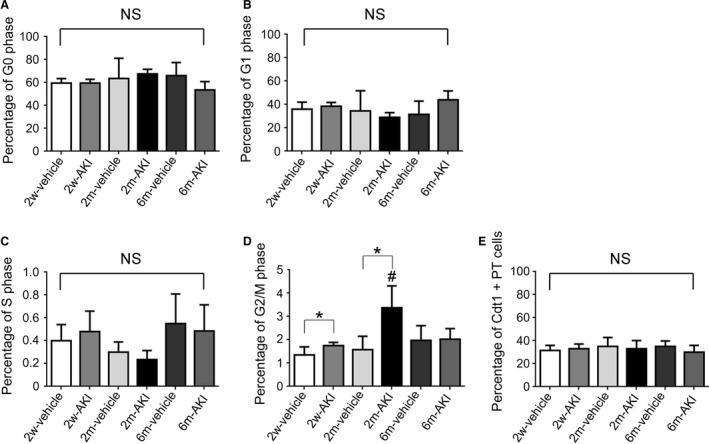
Evaluation of cell cycle status in proximal tubule (PT) cells recovering from acute kidney injury (AKI) before readministration of uranyl acetate (UA). The percentages of G0 (A), G1 (B), S (C), and G2/M phase (D) cells and Cdt1+ cells (E) in the PT of rats pretreated with vehicle and those pretreated with 1 mg/kg of UA are shown at each time point. Data represent the mean ± SD of six rats. *P < 0.05, #*P* < 0.05 versus rats 2 weeks after first treatment. NS, not significant.

**Figure 4:**
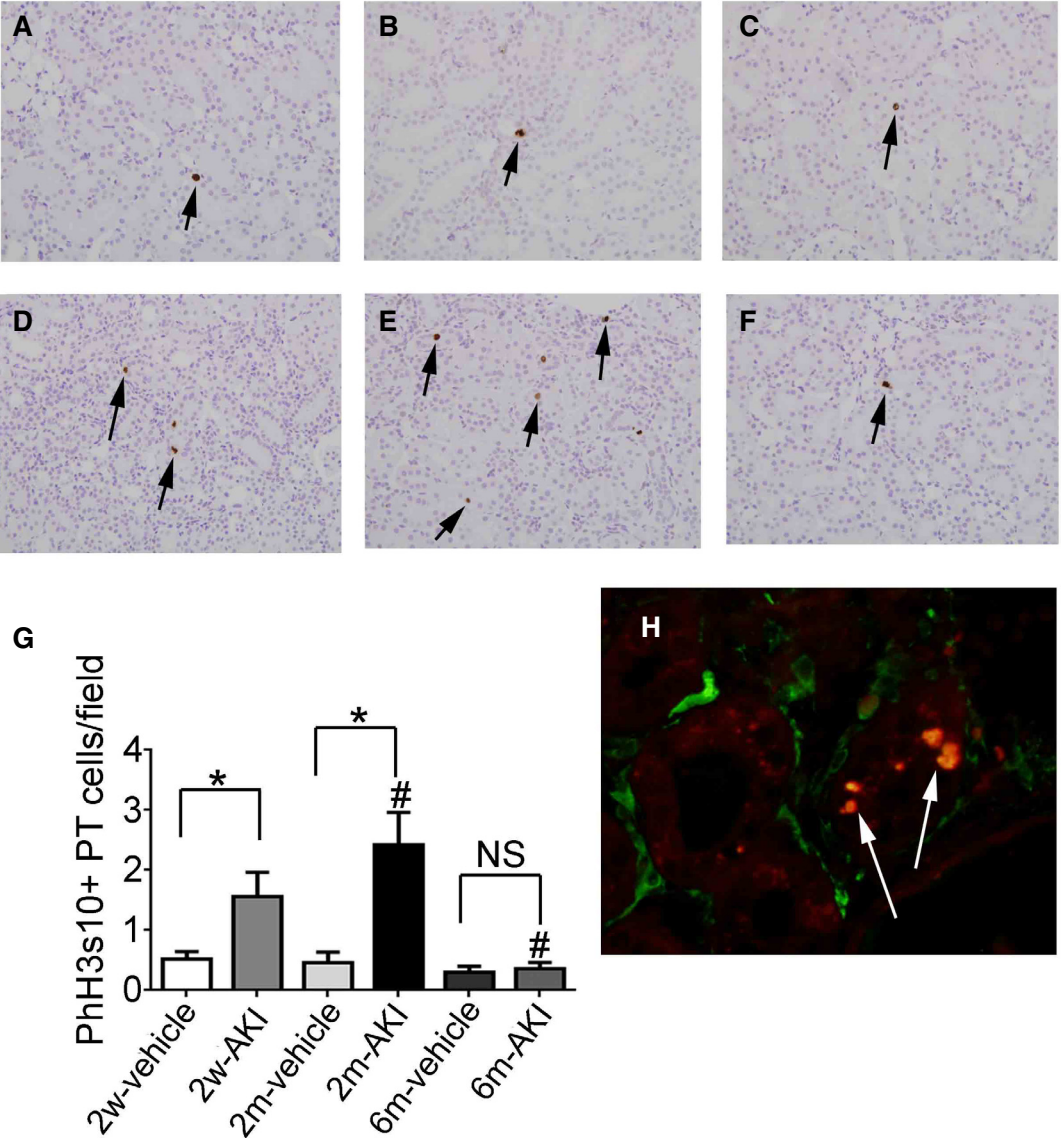
Expression of phosphohistone H3 at serine 10 (PhH3s10) in proximal tubule (PT) cells recovering from acute kidney injury (AKI) before readministration of uranyl acetate (UA). Photomicrographs of PhH3s10‐immunostained (arrows) renal sections from rats pretreated with vehicle (A–C) and with 1 mg/kg of UA (D–F) for 2 weeks (A, D), 2 months (B, E), and 6 months (C, F) after treatment. (G) Temporal changes in the number of PhH3s10+ PT cells in rats pretreated with vehicle or with UA per field. (H) Photomicrograph of double immunostaining with αSMA (green color) and PhH3s10 (arrows, red color) at 2 months after administration of 1 mg/kg of UA. Original magnification, 400×. Data represent the mean ± SD of six rats. *P < 0.05, #*P* < 0.05 versus rats 2 weeks after first treatment. NS, not significant. PhH3s10, phosphohistone H3 at serine 10.

More than 90% of the isolated PT and DT cells were viable in all groups (Table 2) and the purity of isolated PT cells was more than 87% for all groups (Table 3), indicating effective separation of viable PT and DT cells in this study. The percentage of G0, G1, and S phase in isolated PT cells was equivalent in all groups (Fig. 3A–C). The percentage of Cdt1+ cells (G1 phase cells) among isolated PT cells was also equivalent in all groups (Fig. 3E). The percentage of cells in G2/M phase was higher in PT cells of the AKI group than in those of the vehicle group at 2 weeks and 2 months (Fig. 3D); this proportion became equivalent to that in the vehicle group at 6 months. Using flow cytometry and determining the number of PhH3s10+ cells (Fig. 4), which indicates G2/M phase, these results were confirmed for all groups, suggesting that the percentage of G2/M phase cells increased from 2 weeks to 2 months, and returned to basal levels by 6 months after AKI induction.

**Table 2:** Viability of isolated tubular cells

Group	PT (%)			DT (%)		
Time after first treatment	2 weeks	2 months	6 months	2 weeks	2 months	6 months
V	90.3 ± 3.8	95.7 ± 2.9	94.5 ± 1.9	94.6 ± 4.2	99.3 ± 0.2	98.6 ± 1.4
AKI	91.1 ± 3.4	93.6 ± 2.3	94.4 ± 3.3	94.1 ± 4.0	97.4 ± 1.0	97.4 ± 2.7

Data represent the mean ± SD. There were no differences in the viability among all groups. PT, proximal tubule; DT, distal tubule; AKI, pretreated with 1 mg/kg of uranyl acetate; V, rats pretreated with vehicle.

**Table 3:** Purity (megalin positivity) of isolated tubular cells

Group	PT (%)			DT (%)		
Time after first treatment	2 weeks	2 months	6 months	2 weeks	2 months	6 months
V	91.7 ± 2.5	92.2 ± 4.2	89.1 ± 2.8	7.9 ± 3.7	9.9 ± 3.8	10.5 ± 3.0
AKI	91.3 ± 2.7	91.8 ± 2.2	87.0 ± 5.9	10.5 ± 4.5	10.3 ± 3.6	10.0 ± 3.9

Data represent the mean ± SD. There were no differences in the megalin positivity among all PT groups or DT groups. PT, proximal tubule; DT, distal tubule; AKI, pretreated with 1 mg/kg of uranyl acetate; V, rats pretreated with vehicle.

**Figure 5:**
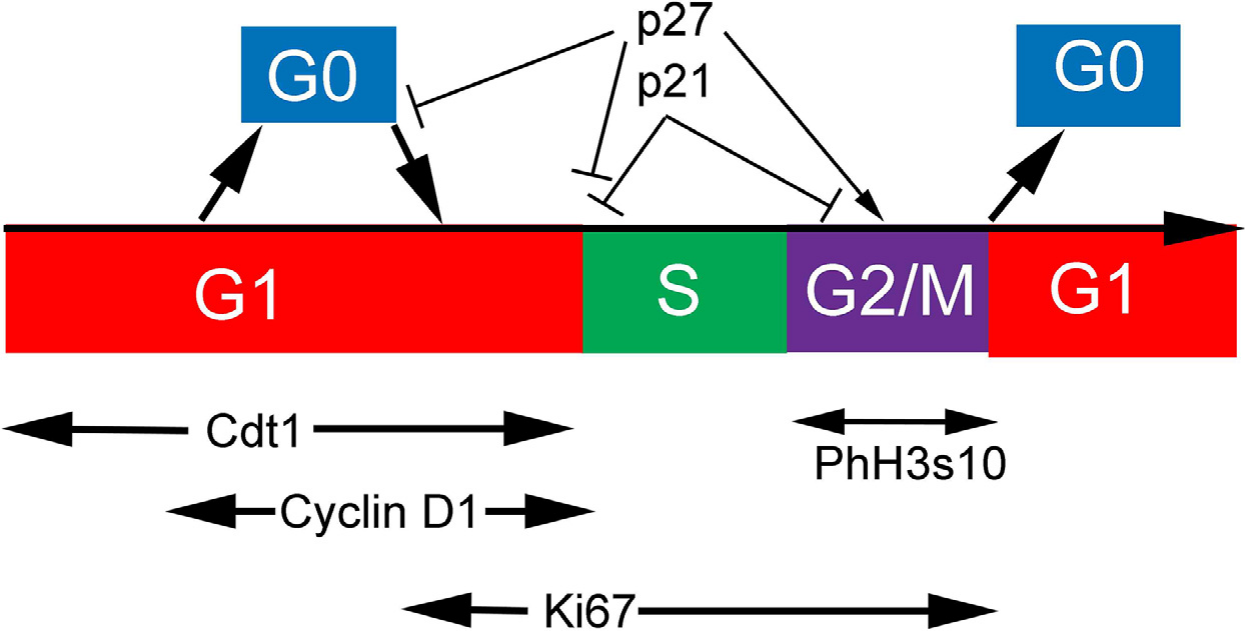
Schematic drawing of the cell cycle phases with respective cyclin‐dependent kinase inhibitors and cell cycle markers. Cdt1 is a marker of the entire G1 phase. Cyclin D1 is a mid–late G1 phase marker. Ki67 is a late G1 to G2/M phase marker. Phosphorylated histone H3 at serine 10 is a G2/M phase marker. p21 associates with G1–S and G2/M phase transition. p27 associates with G0–G1, G1–S, and G2/M phase transition. PhH3s10, phosphohistone H3 at serine 10.

**Figure 6:**
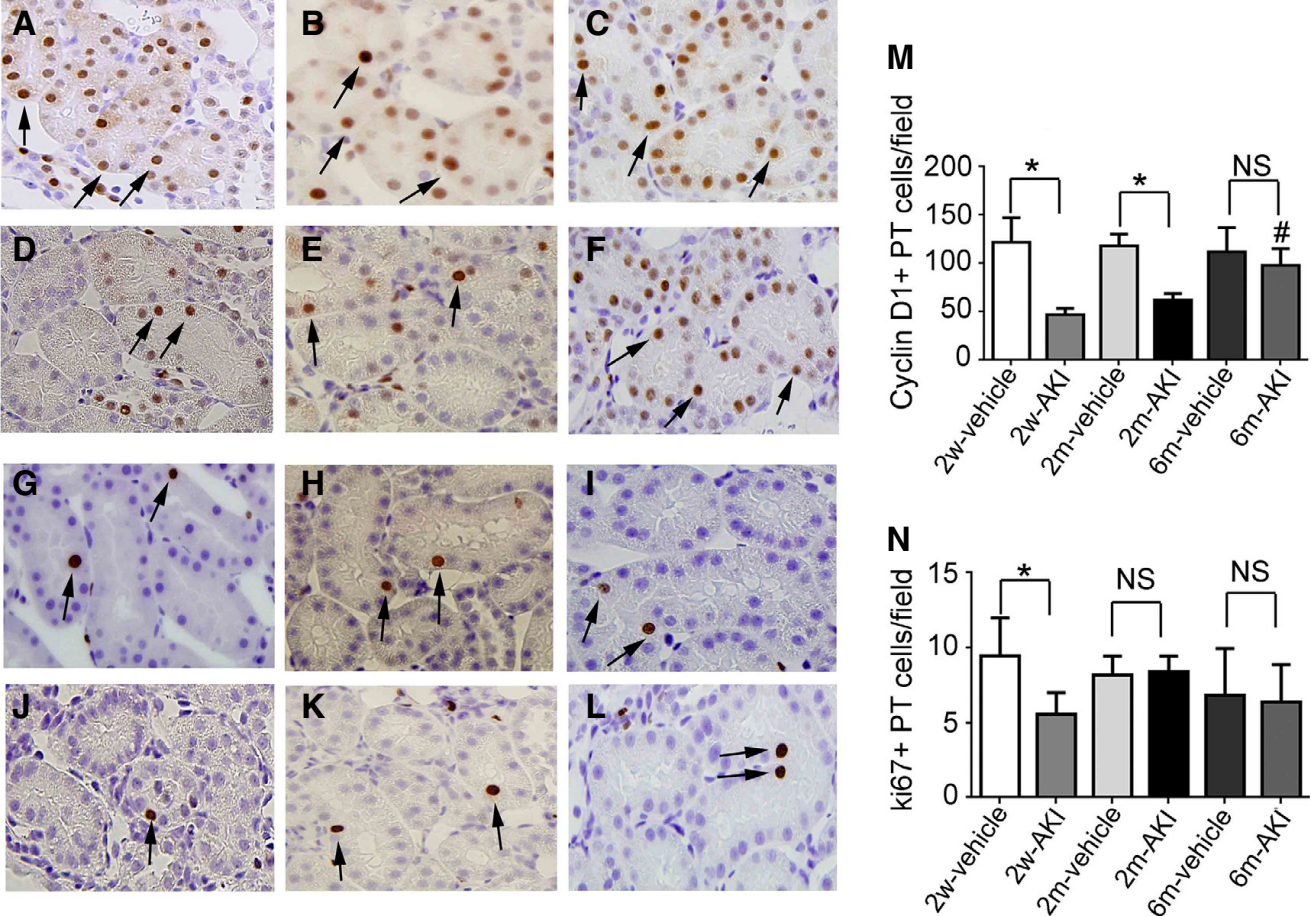
Expression of cyclin D1 and Ki67 in proximal tubule (PT) cells recovering from acute kidney injury (AKI) before readministration of uranyl acetate (UA). Photomicrographs of cyclin D1‐immunostained (arrows) renal sections from rats pretreated with vehicle (A–C) or with 1 mg/kg of UA (D–F) for 2 weeks (A, D), 2 months (B, E), and 6 months (C, F) after treatment. Photomicrographs of Ki67‐immunostained (arrows) renal sections from rats pretreated with vehicle (G–I) and with 1 mg/kg of UA (J–L) for 2 weeks (G, J), 2 months (H, K), and 6 months (I, L). Temporal changes in the number of cyclin D1+ (M) or Ki67+ (N) PT cells per field in rats pretreated with vehicle or with UA. Original magnification, 400×. Data represent the mean ± SD of six rats. *P < 0.05, #*P* < 0.05 versus rats 2 weeks after first treatment. NS, not significant.

**Figure 7:**
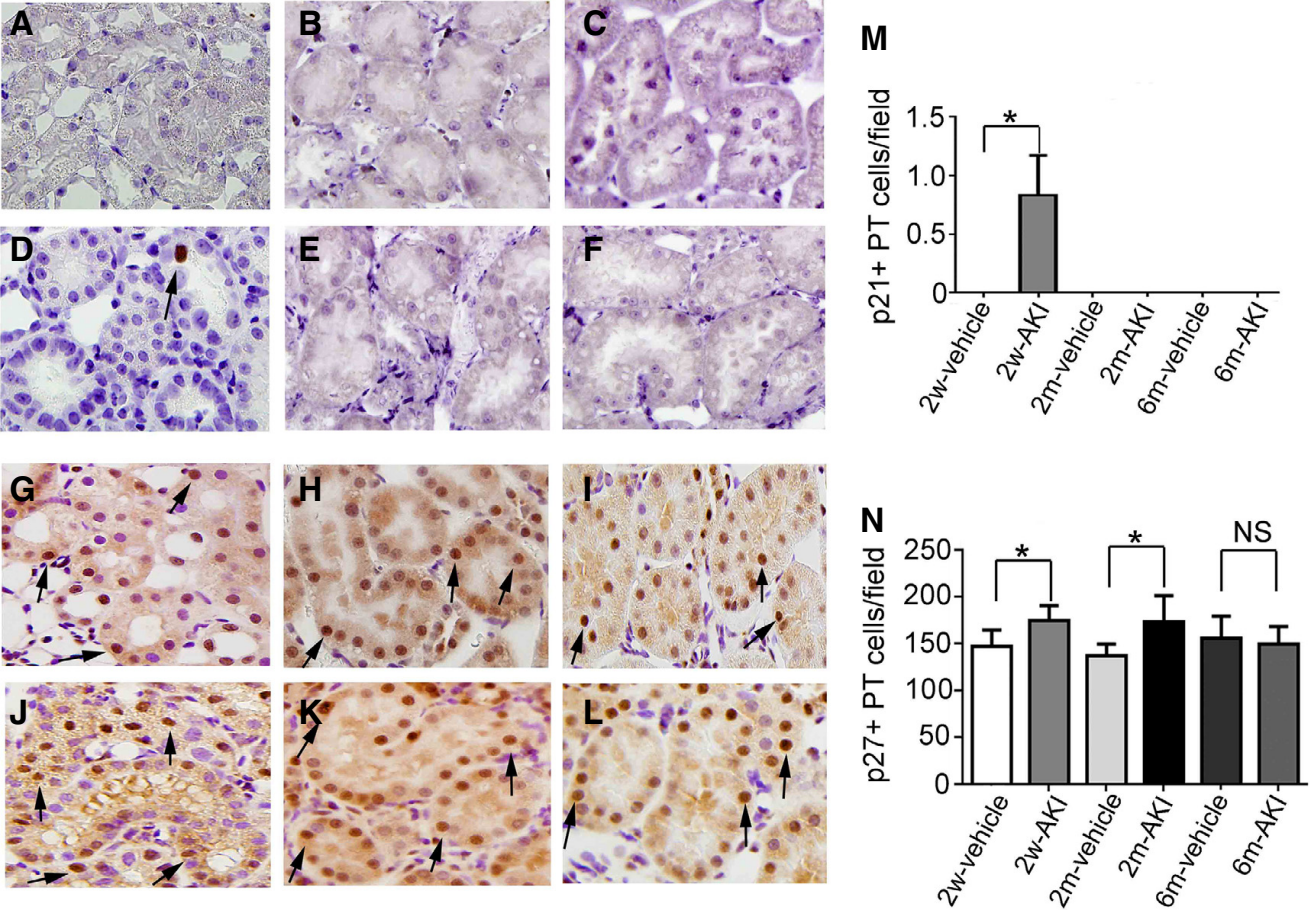
Expression of p21 and p27 in proximal tubule (PT) cells recovering from acute kidney injury (AKI) before readministration of uranyl acetate (UA). Photomicrographs of p21‐immunostained (arrow) renal sections from rats pretreated with vehicle (A–C) or with 1 mg/kg of UA (D–F) for 2 weeks (A, D), 2 months (B, E), and 6 months (C, F) after treatment. Photomicrographs of p27‐immunostained (arrows) renal sections from rats pretreated with vehicle (G–I) and with 1 mg/kg of UA (J–L) for 2 weeks (G, J), 2 months (H, K), and 6 months (I, L) after treatment. Temporal changes in the number of p21+ (M) or p27+ (N) PT cells per field in rats pretreated with vehicle with UA. Original magnification, 400×. Data represent the mean ± SD of six rats. *P < 0.05. NS, not significant.

We used cyclin D1 and Ki67 as cell cycle markers. A simplified schematic diagram is provided showing the relationship of cyclin D1, Ki67, and the CDKIs p21 and p27 with cell cycle phase (Fig. 5). The number of cyclin D1+ PT cells was significantly lower at day 0 in the 2w‐ and 2m‐AKI groups than in the 2w‐ and 2m‐vehicle groups, respectively. However, at day 0 in the 6m‐AKI group, this number was comparable to that in the 6m‐vehicle group (Fig. 6A–F and M). The number of Ki67+ PT cells was significantly lower at day 0 in the 2w‐AKI group than in the 2w‐vehicle group. In contrast, this number at day 0 in the 2m‐ and 6m‐AKI groups was comparable to that in 2m‐ and 6m‐vehicle groups, respectively (Fig. 6G–L and N). These results suggest that both cyclin D1 and Ki67 expression normalized by 6 months after UA‐induced AKI. There were significantly more p21+ cells in the PT at day 0 in the 2w‐AKI group than in the 2w‐vehicle group, but this difference was lost at 2 and 6 months (Fig. 7A–F and M). In contrast, the number of p27+ PT cells was significantly higher at day 0 in the 2w‐ and 2m‐AKI groups than in the 2w‐ and 2m‐vehicle groups, respectively; however, this number in the 6m‐AKI group became comparable to that in the 6m‐vehicle group (Fig. 7G–L and N), suggesting that the expression of both p21 and p27 normalized by 6 months after UA‐induced AKI.

### Phenotypic recovery after injury in PT cells

**Figure 8:**
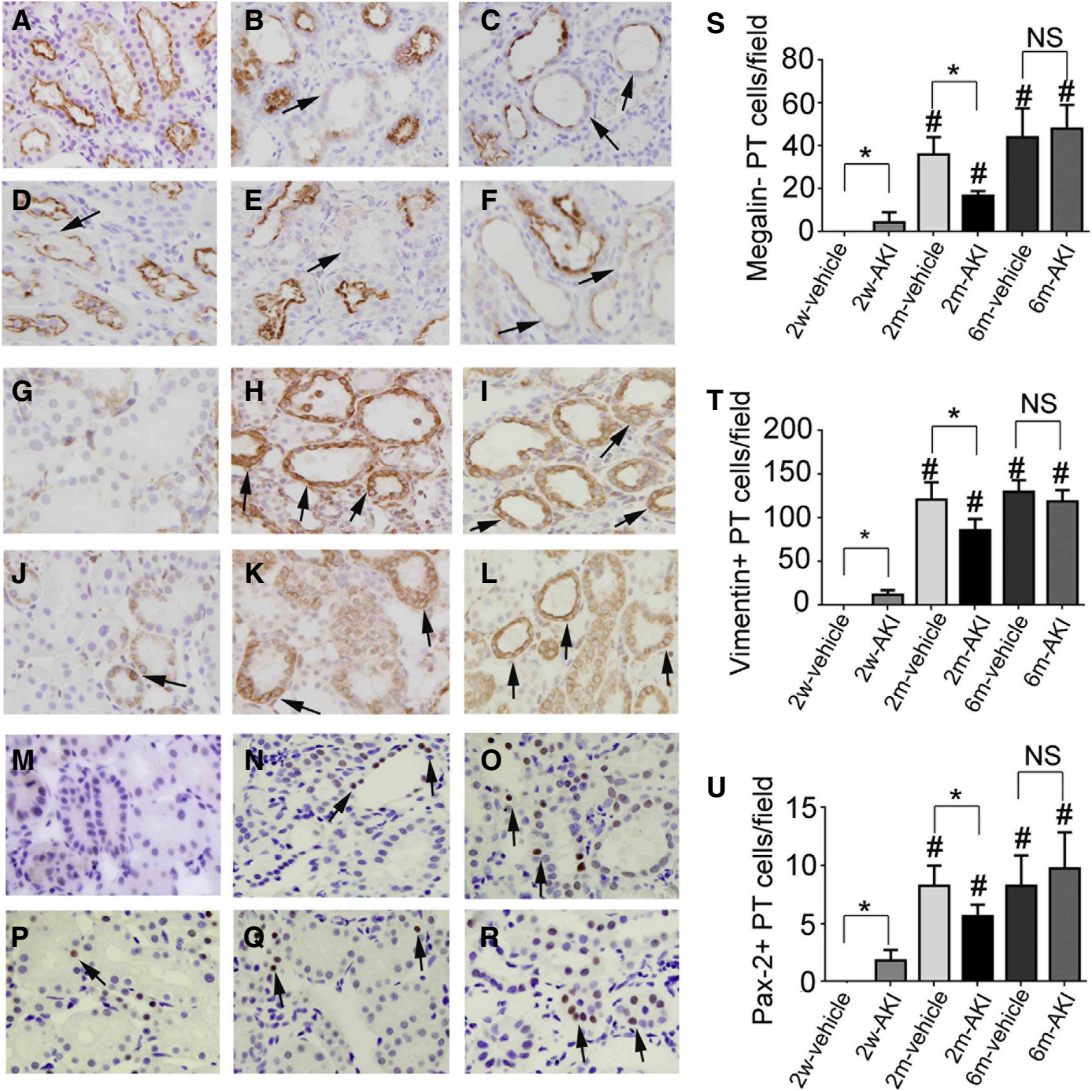
Dedifferentiation of proximal tubule (PT) cells recovering from acute kidney injury (AKI) after readministration of uranyl acetate (UA). Photomicrographs of megalin‐immunostained renal sections from rats pretreated with vehicle (A–C) or with 1 mg/kg of UA (D–F) for 2 weeks (A, C), 2 months (B, D), and 6 months (C, E), 14 days after readministration of UA. Photomicrographs of vimentin‐immunostained (arrows) renal sections from rats pretreated with vehicle (G–I) or with 1 mg/kg of UA (J–L) for 2 weeks (G, J), 2 months (H, K), and 6 months (I, L), 14 days after readministration of UA. Photomicrographs of Pax2‐immunostained (arrows) renal sections from rats pretreated with vehicle (M–O) or rats pretreated with 1 mg/kg of UA (P–R) for 2 weeks (M, P), 2 months (N, Q), and 6 months (O, R), 14 days after readministration of UA. Temporal changes in the number of megalin− (S), vimentin+ (T), or Pax2+ (U) PT cells per field 14 days after readministration of UA. Original magnification, 400×. Data represent the mean ± SD of six rats. *P < 0.05, #*P* < 0.05 versus rats 2 weeks after first treatment. NS, not significant.

Almost all PT cells expressed megalin (a marker of PT cell differentiation) just before the second treatment (day 0) in all AKI groups, whereas no PT cells expressed vimentin or Pax‐2 (markers of PT cell dedifferentiation) at day 0 in all AKI groups (data not shown). This indicates that cells recovering from AKI returned to a mature PT state as early as 2 weeks postchallenge. The number of megalin−, vimentin+, or Pax‐2+ PT cells (dedifferentiated cells) increased significantly at day 14 in all groups, except for the 2w‐vehicle group, after the second insult. The number of megalin−, vimentin+, or Pax‐2+ PT cells at day 14 in the 2m‐vehicle and 6m‐vehicle groups was higher than that in the 2w‐vehicle group, respectively (Fig. 8), suggesting that redifferentiation of PT cells was delayed in older rats in the 2m‐ and 6m‐vehicle groups after UA insult, when compared to that in younger rats in the 2w‐vehicle group.

Vimentin+ or Pax‐2+ PT cells were sparsely distributed at day 14 in the 2w‐AKI group, suggesting that a certain number of PT cells did not accomplish redifferentiation after the second insult. The number of megalin−, vimentin+, or Pax‐2+ PT cells increased significantly at day 14 in the 2m‐AKI group, but this value was lower than that in the 2m‐ and 6m‐vehicle groups. The number of megalin−, vimentin+, or Pax‐2+ PT cells at day 14 in the 6m‐AKI group was not different from that in the 6m‐vehicle group (Fig. 8), suggesting that PT cells in the 6m‐AKI group were comparable to those in the 6m‐vehicle group in terms of the kinetics of phenotypic change after UA insult.

**Figure 9:**
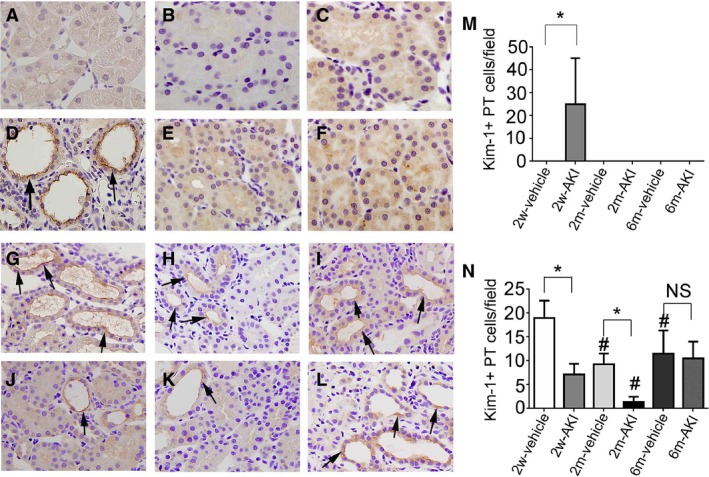
Histological changes in proximal tubules (PTs) recovering from acute kidney injury (AKI) before and after readministration of uranyl acetate (UA). Photomicrographs of Kim‐1‐immunostained (arrows) renal sections from rats pretreated with vehicle (A–C) or with 1 mg/kg of UA (D–F) for 2 weeks (A, C), 2 months (B, D), and 6 months (C, E) after treatment. Photomicrographs of Kim‐1‐immunostained (arrows) renal sections from rats pretreated with vehicle (G–I) or with 1 mg/kg of UA (J–L) 14 days after readministration of UA in rats for 2 weeks (G, J), 2 months (H, K), and 6 months (I, L) after the first treatment. (M, N) Temporal changes in the number of Kim‐1+ PT cells per field before (M) and 14 days after (N) readministration of UA. Data represent the mean ± SD of six rats. *P < 0.05, #*P* < 0.05 versus rats 2 weeks after first treatment. NS, not significant.

Some PT cells still expressed Kim‐1, a marker of PT injury, at day 0 in the 2w‐AKI group. However, Kim‐1+ PT cells were not found at day 0 in the 2m‐ and 6m‐AKI groups (Fig. 9A–F and M), indicating that cells had recovered from UA‐induced PT injury by 2 months.

The number of Kim‐1+ PT cells at day 14 in the 2m‐ and 6m‐vehicle groups was lower than that in the 2w‐vehicle group. The number of Kim‐1+ PT cells was lower at day 14 in the 2w‐ and 2m‐AKI groups than in the 2w‐ and 2m‐vehicle groups, respectively. However, this number at day 14 in the 6m‐AKI group became comparable to that in the 6m‐vehicle group (Fig. 9G–L and N), suggesting that the degree of PT injury after UA insult might be similar between the 6m‐AKI and 6m‐vehicle groups.

### Transient accumulation of interstitial collagen with AKI

**Figure 10:**
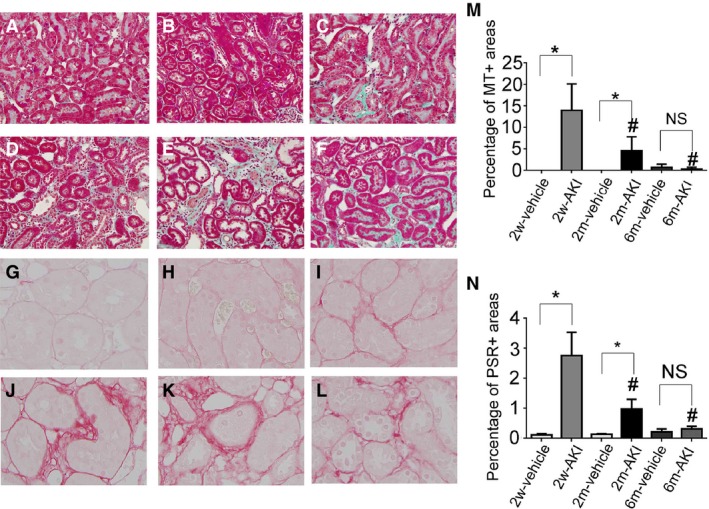
Fibrosis and collagen deposition in the interstitium recovering from acute kidney injury (AKI). Photomicrographs of Masson's trichrome‐stained renal sections from rats pretreated with vehicle (A–C) or with 1 mg/kg of uranyl acetate (UA) (D–F) for 2 weeks (A, D), 2 months (B, E), and 6 months (C, F) after treatment. Photomicrographs of picrosirius red‐stained renal sections from rats pretreated with vehicle (G–I) and with 1 mg/kg of UA (J–L) for 2 weeks (G, J), 2 months (H, K), and 6 months (I, L) after treatment. (M) Temporal changes in the area of tubulointerstitial fibrosis evaluated as Masson's trichrome+ areas per field. (N) Temporal changes in the picrosirius red+ area. A–F, Original magnification 100×. G–L, Original magnification 400×. Data represent the mean ± SD of 6 rats. *P < 0.05, #*P* < 0.05 versus rats 2 weeks after first treatment. NS, not significant, MT, Masson's trichrome, PSR, picrosirius red.

Tubulointerstitial fibrosis, evaluated by Masson's trichrome staining and picrosirius red staining, was mainly found around the PTs. The percent area that stained positive for both agents in the cortex and OSOM at day 0 in the 2w‐ and 2m‐AKI groups was higher than that in the 2w‐ and 2m‐vehicle groups, respectively. However, this value at day 0 in the 6m‐AKI group was comparable to that in the 6m‐vehicle group (Fig. 10). These findings indicate that UA‐induced AKI resulted in the transient accumulation of interstitial collagen surrounding the PTs.

αSMA+ cells surrounding the PT appeared transiently at day 0 in the 2w‐ and 2m‐AKI groups and virtually disappeared at day 0 in the 6m‐AKI group (data not shown). Double immunostaining of PhH3s10 and αSMA showed that the αSMA+ areas were found in the vicinity of PhH3s10+ PT cells (Fig. 4H), suggesting that G2/M phase PT cells are associated with αSMA+ myofibroblasts, which might produce interstitial collagen.

## Discussion

In this study, we found that acquired cytoresistance in PT cells is limited to the period before normalization of cell number and subcellular molecular integrity, from a hyperplastic state to baseline levels after UA‐induced AKI. A summary of the sequence of changes in terms of the degree of cytoresistance and the associated factors, found in this study, is shown in Table 4.

**Table 4:** Summary of serial changes in the degree of cytoresistance in PT cells, the cell number of PT, YAP/survivin expression, cell cycle status, expression of G1 phase‐mediated factors in PT cells and interstitial fibrosis after UA administration

Time after primary UA administration	2 weeks	2 months	6 months
Cytoresistance	↑↑	↑	→
Cell number	↑↑	↑	→
YAP expression	↑↑	↑	→
Survivin expression	↑↑	↑	→
G0 phase	→	→	→
G1 phase	→	→	→
S phase	→	→	→
G2/M phase	↑	↑↑	→
Cyclin D1	↓	↓	→
Ki67	↓	→	→
p21	↑	→	→
p27	↑	↑	→
Interstitial fibrosis	↑↑	↑	→

→, no significant change; ↑, increase or upregulation; ↓, decrease or downregulation from steady‐state conditions.

In accordance with our previous report (Sano et al. 2000), in the present study, the number of PT cells increased over that of the baseline at 2 weeks, then gradually returned to normal by 6 months after the induction of AKI. YAP, an effector of the Hippo signaling pathway, is known to control organ size by modulating cell growth (Dong et al. 2007; Yu and Guan 2013). Overexpression of YAP can induce hyperplasia, and its cancellation regresses organ size (Dong et al. 2007). It has been reported that YAP is activated during tissue regeneration after hepatectomy, and that nuclear YAP was transiently activated even after the liver reached prehepatectomy size (Grijalva et al. 2014); this was shown to result in liver hyperplasia following hepatectomy (Islami et al. 1959). In the present study, transient overexpression of nuclear YAP was also found in PT cells that had recovered from AKI. The increased number of YAP+ PT cells was associated with an increased number of cells in cross‐sectional PT, suggesting that the overexpression of YAP contributes to hyperplasia in the PT, similar to that observed after hepatectomy. The transient increase in YAP in PT cells that had recovered from AKI might be explained by the fact that YAP functions to increase and/or control the number of PT cells during recovery to compensate for injury after insult.

Interestingly, the overexpression of YAP in PT cells was associated with acquired cytoresistance after the second UA insult. Because survivin, a member of the inhibitor of apoptosis family, was reported to be a downstream mediator of YAP (Dong et al. 2007), the overexpression of YAP is thought to result in the evasion of apoptosis (Dong et al. 2007; Campbell et al. 2013). Indeed, since the number of YAP+ cells in PT was positively correlated with the number of survivin+ cells, it is conceivable that YAP mediates survivin upregulation, contributing to cytoprotection. We and Mehendale et al. (Korrapati et al. 2005, 2006; Sun et al. 2010, 2011; Fujikura et al. 2013) have demonstrated that former exposure of low dose of nephrotoxicant induced the facilitation of proliferation in response to subsequent nephrotoxic dose of nephrotoxicant, resulting in attenuation of AKI. These reports suggest that the facilitation of tubular cell proliferation may be linked to cytoprotection. It has been reported that the overexpression of survivin induced acceleration of proliferation in cancer cells (Singh et al. 2015), and that survivin had a crucial role for the functional and structural recovery of the kidney from ischemia–reperfusion AKI model and of a mouse renal proximal tubular cell line (Chen et al. 2013). The protective role of survivin in cisplatin‐ and folic acid‐induced AKI has been reported previously (Kindt et al. 2008). Therefore, it might be possible that there is a universal relationship between cytoresistance of tubular cells and cellular recovery with YAP/survivin expression after AKI.

As previously reported (Iwakura et al. 2016), in the present study, the PT phenotype returned to a redifferentiated mature state (megalin+/vimentin−/pax2−) as early as 2 weeks after the first UA insult. Meanwhile, recovery from tubular cell injury, evaluated by Kim‐1 expression, occurred as early as 2 months after the first UA insult. Based on these results, it appeared as if PT cells were normalized as early as 2 months after UA insult. However, the expression of cell cycle‐mediating factors did not return to normal at that point. Recently, we reported that acquired cytoresistance was associated with enhanced G1 arrest via the modulation of cell cycle‐regulating factors including cyclin D1, p21, and p27 (Iwakura et al. 2016). In accordance with our previous report, the percentage of cells exhibiting early G1 arrest (cyclin D1−), among all G1 phase cells, and the number of p27+ cells in the PT were higher in the AKI groups than in the vehicle groups with cytoresistance, and this normalized when cytoresistance was lost. An increase in p21 occurred at day 0 in the 2w‐AKI group, but not in the 2m‐ and 6m‐AKI groups. It has been reported that p21 overexpression is renoprotective (Megyesi et al. 2002). Thus, the partial attenuation of acquired resistance in the 2m‐AKI group might be associated with the normalization of p21 levels. As YAP expression and PT cell numbers were normalized, cell cycle‐mediating factors such as cyclin D1, p21, and p27 were also normalized. However, it has been reported that YAP overexpression induces an increase in cyclin D1 and a reduction in p21/p27, accelerating the proliferation in human corneal endothelial cells (Hsueh et al. 2015).

In the present in vivo AKI model, it is hypothesized that there is a mixture of p21/p27+ G1 arrested cells and YAP+ proliferating cells, which depends on the degree and/or phase of injury after the induction of AKI. Therefore, making interpretations based on only the number of cells expressing cell cycle‐regulating factors or YAP positivity is associated with limitations. Cell cycle‐mediating factors are also controlled by several factors other than YAP (Gérard and Goldbeter 2014). Moreover, PT cell fate regulated by YAP expression might be influenced by kidney size relative to body weight in rats of different ages (Melk et al. 2003).

It has been reported that PT cells that have arrested at the G2/M phase after the induction of AKI actively release profibrotic cytokines beyond the tubular basement membrane, which stimulates proliferation and collagen production in interstitial fibroblasts (Yang et al. 2010). In the present study, the percent of G2/M phase cells in PTs in the 2w‐ and 2m‐AKI groups was higher than that in the 2w‐ and 2m‐vehicle groups, respectively, without an increased proportion of S phase cells, suggesting that G2/M arrest was sustained until 2 months after the induction of AKI. αSMA+ cells were transiently observed around the PT and were in close proximity to G2/M phase PT cells, suggesting that G2/M‐arrested PT cells can induce collagen production in αSMA+ myofibroblasts in the UA‐induced AKI model. We found that fibrosis, evaluated by Masson's trichrome staining, or interstitial collagen deposition, assessed by picrosirius red staining, after UA‐induced AKI was reversible and was associated with the transient appearance of αSMA+ cells. The reversibility of fibrosis was previously described in the liver (Liu et al. 2013) and in a unilateral ureteral obstruction model, based on the reversal of disease etiology (Cochrane et al. 2005). In the present AKI model, we confirmed that interstitial fibrosis is spatiotemporally associated with G2/M phase PT cells after the induction of AKI. In addition, it was reported that nuclear YAP expression was associated with production of profibrogenic cytokines in the human HK‐2 proximal tubular cell line (Xu et al. 2016), thus regression of YAP might also contribute to the reversal of fibrosis. Moreover, other cell types such as infiltrating macrophage as a profibrogenic cytokine producing cell and pericyte as a collagen producing cell have been reported as associated factors of interstitial fibrosis after AKI (Chen et al. 2011; Khairoun et al. 2013; Kim et al. 2015). Regression of renal interstitial fibrosis is also an important issue to prevent the progression of AKI to chronic kidney disease in clinical settings. Therefore, further studies on the mechanisms underlying the reversal of fibrosis after AKI are necessary in relation to the above factors.

In conclusion, upregulation of the Hippo‐YAP signaling pathway in PT cells and its downstream mediator survivin were associated with cytoresistance in PTs, probably due to the antiapoptotic effect of survivin, over the course of tubular recovery after AKI. Overexpression of YAP induced hyperplasia in PTs, and when this subsided, the number of PT cells with enhanced cell cycle arrest was gradually diminished. The current study demonstrates that PT cytoresistance occurs until the completion of remodeling, in terms of cell number and subcellular integrity. Since this study is observational, we may caution to draw any mechanistic conclusion from these experiments.

## Conflict of Interest

None declared.
